# Cervical and preauricular lymphadenopathies as atypical manifestations in the setting of COVID-19: a case report

**DOI:** 10.2217/fvl-2021-0074

**Published:** 2022-02-11

**Authors:** Alireza Ala, Solomon Habtemariam, Samad Shams Vahdati, Aysa Rezabakhsh

**Affiliations:** ^1^Emergency & Trauma Care Research Center, Tabriz University of Medical Sciences, Tabriz, 5166615573, Iran; ^2^Pharmacognosy Research Laboratories & Herbal Analysis Services UK, University of Greenwich, Chatham-Maritime, Kent, ME4 4TB, UK; ^3^Cardiovascular Research Center, Tabriz University of Medical Sciences, Tabriz, 5166615573, Iran

**Keywords:** COVID-19, herpes-like skin manifestation, lymphadenopathy, SARS-CoV-2

## Abstract

Besides the common symptoms in COVID-19, it has been thought to be a more imperative measure to identify the extraordinary manifestations of the illness, which would be more helpful to improve clinical management. In the current report, a 39-year-old woman and a 44-year-old man showed reactive cervical and preauricular lymphadenopathies, respectively, upon a range of the common symptoms of the disease. Interestingly, none of them showed the symptoms of lower respiratory tract infection as well. Notably, a herpes-like skin lesion was also observed on the right lower eyelid in one of the positive patients.

Acknowledged by the WHO as a global pandemic, COVID-19 has brought up an immense socioeconomic burden to the world population [1]. Following infection induced by the SARS-CoV-2, the disease symptoms extend from asymptomatic to a wide array of mild and severe forms [2,3]. Dry cough, headache, fever, sore throat, fatigue, dyspnea, the loss of the smelling/tasting sense and myalgia are considered as common manifestations that can further develop into a complicated form with poor prognosis, named acute respiratory distress syndrome [4].

Of note, underlying comorbidities, including hypertension, aging, cancers, immunocompromised conditions and diabetes, are considered major factors that increase the risk of COVID-19 [5–7]. Alongside the reverse transcriptase-PCR (RT-PCR) assay, spiral chest computed tomography (CT) scan with the bilateral ground-glass opacity manifestation is described as the reliable and integral part of the COVID-19 diagnostic criteria in the early stage of the illness [8,9]. In addition, according to the updated guidelines, the ‘SARS-CoV-2-specific immunoglobulin (Ig)M and IgG antibodies’ could be considered reliable tools for distinguishing positive patients infected by COVID-19 and those in the convalescence period with a negative PCR result [10].

The current therapeutic strategies for COVID-19 refer to appropriate and timely administration of both antiviral (e.g., remdesivir) [11] and glucocorticoid (e.g., dexamethasone) [12] medications in viral and inflammatory phases, respectively [10]. Moreover, the medications with antithrombotic effects and some monoclonal antibodies, for example, tocilizumab with direct IL-6 inhibitory effect, are also recommended in critically patients admitted to the intensive care unit [13].

However, understanding the unusual clinical manifestations could be more helpful for clinicians to manage the disease better. Despite cervical lymphadenopathy (CLA) multiple etiologies, including malignancies, mycobacterial infections [14,15], Epstein–Barr virus related mononucleosis [16], toxoplasmosis and HIV [17], it has been observed in the non severe form of COVID-19, as well. Besides, some cutaneous manifestations were also reported following the SARS-CoV-2 infection, including rash [18], petechiae, pernio-like acral lesions [19], urticaria [20,21], macular erythema, chilblain-like or vesicular eruption and papulosquamous eruption [21–23]. The present case series mainly uncoverd some uncommon manifestations in confirmed COVID-19 patients.

## Case presentation

### Case 1

**Figure 1.**
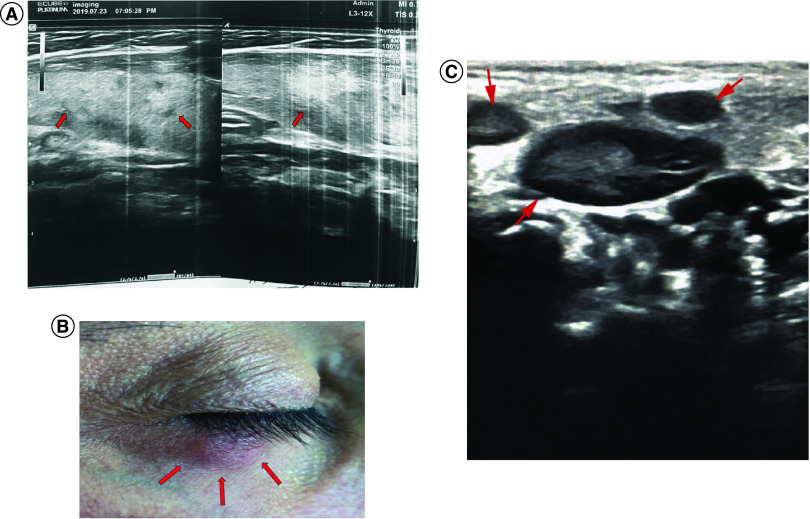
Examination of the cervical and preauricular lymphadenopathies, as well as skin lesion. **(A)** The ultrasound imaging in terms of the CLA detection in case 1 (maximum size = 14 mm in diameter, which has been shown with red arrays). **(B)** A herpes-like skin lesion represented on the right lower eyelid, as a cutaneous manifestation of COVID-19 in case 2. **(C)** The ultrasound imaging for inflammation-derived preauricular lymphadenopathy detection with preserved fatty hilum in case 2. The SAD regarding cortical thickness was estimated between 10–14 mm, which has been shown with red arrays. CLA: Cervical lymphadenopathy; SAD: Short axis diameter.

A 39-year-old woman with a medical history of insulin-dependent diabetes, who was in prolonged close contact with a COVID-19-positive subject in an indoor setting, was referred to the healthcare center with chief complaints of fever, sore throat, dizziness, headache, painful enlarged cervical lymph nodes, renal pain, frequent nocturnal urination, heart palpitation and progressive asthenia during the previous month. No pathological changes in thyroid lobes were detected following the thyroid ultrasound examination. However, numerous hypoechoic nodules with a maximum size of 14 mm were detected on the right side of the neck region (Figure 1A).

The paraclinical examinations also showed an increased level of procalcitonin ([PCT], >2 ng/ml), currently indicated as COVID-19 severity predictor, hemoglobin A1C (8.7%), remarkable elevated erythrocyte sedimentation rate (patient erythrocyte sedimentation rate [ESR] = 53 vs reference ESR ≤ 20 mm/h) and slight neutrophilia (70.1%). Next, she was hospitalized due to uncontrolled hyperglycemia and upper respiratory tract infection for 48h, and empirically received broad-spectrum antibiotic meropenem, 1 g iv., twice a day (b.i.d.) for intensive monitoring of the concomitant septicemia and insulin glargine (Lantus^®^) and insulin aspart (Novorapid^®^), as well as a fixed-dose combination antidiabetic medication (empagliflozin/metformin hydrochloride, 5/1000 mg, orally), were also applied for blood glucose adjustment. Following septicemia, a chest CT scan was also performed due to the sudden loss of smell sense which increased the possibility of COVID-19 infection that appeared upon the unresolved mild fever, myalgia and dizziness after 2 days of admission. However, the results of CT scan imaging did not show any lower respiratory tract infection.

After being discharged from the hospital, she constantly complained of painful CLA, renal pain and acute myalgia. In addition, the results of laboratory test with 5-day interval still showed the high level of erythrocyte sedimentation rate (60 mm/h), remarkably elevated platelet count and PCT, along with the concentration of IgG higher than the optimal cut off value (17.6 Au/ml) and the normal rate of IgM (1.6 Au/ml), which finally confirmed the recent COVID-19 infection in this patient following the positive result of RT-PCR (Table 1).

**Table 1.** The results of complete blood count and serologic tests of the case 1.

CBC	Result	Unit	Ref range	Differential	Results (%)
RBC HGB HCT MCV MCH MCHC RDW-CV RDW-SD PLT MPV PDW PCT P-LCC P-LCR	3.91 **9.8** **31.1** **79.6** **25.1** **31.5** 12.7 38.8 **493** 7.6 15.8 **0.375** **95** 19.2	×10^6^/ul g/dl % fl pg g/dl % 10^3^/Ul fL ng/ml ×10^9^/l %	3.5-5.0 12.0-16.0 35.0-45.0 80.0-100.0 27.0-34.0 32.0-36.0 11.0-16.0 35.0-56.0 140-450 6.5-12.0 9.0-17.0 ≤0.15 30-90 11.0-45.0	Neutrophils Lymphocytes Monocytes Eosinophils Basophils	**70.1** 23.3 4.6 1.7 0.3

**Serologic test**					
ESR 1st h C-reactive protein	**60** 1.5	mm/h mg/l	<20 0-10		

The measurement of each parameter is calculated based on a calibrated device or using related kits, the values out of reference rate are reported with bold format.

ESR: Erythrocyte sedimentation rate; HGB: Hemoglobin; HCT: Hematocrit; MCH: Mean corpuscular hemoglobin; MCHC: Mean cell hemoglobin concentration; MCV: Mean corpuscular volume; MPV: Mean platelet volume; P-LCC: Platelet large cell count; P-LCR: Platelet larger cell ratio; PCT: Procalcitonin; PDW: Platelet distribution width; PLT: Platelet; RBC: Red blood cell; RDW-CV: Red blood cell distribution width-CV; RDW-SD: Red blood cell distribution width-SD.

### Case 2

In a 44-year-old man with a medical history of controlled hypertension (by daily taking Inderal 10 mg and Valsacor^®^ 80 mg), a herpes-like skin lesion in the inferolateral side of the right lower eyelid was observed on 25 October 2020, which was diagnosed by an experienced dermatologist (Figure 1B). He was frequently in close contact with severe and nonsevere COVID-19 patients in a specialized medical center under stressful conditions. He also experienced general asthenia and headache on 30 October 2020, accompanied by tangible and painful preauricular lymphadenopathy following the clinical examination in the anterior part of the right tragus on 1 November 2020. As shown in Figure 1C, the result of ultrasound imaging overtly showed a preserved relatively large-sized fatty hilum and cortical thickness of lymph node with short-axis diameter ≥10 mm.

One day later, the loss of taste and smell senses, as well as nasal congestion, also appeared. However, similar to case 1, he did not present any symptoms of lower respiratory tract infection. Following the pharyngeal swab test and performing RT-PCR diagnostic assay, he was also definitively identified as a SARS-CoV-2-positive patient. Of note, LA was resolved upon the recovery from SARS-CoV-2 infection in both patients within 1 month.

## Discussion & conclusion

In this case series, we aimed to present some uncommon manifestations in COVID-19-positive patients during the incubation period of COVID-19 with the appearance of cervical and preauricular LAs, as well as herpes-like skin lesions, for the first time in Iran. Given that inflammatory reactions robustly elevate during SARS-CoV-2 infection (cytokine storm), it has been proposed that a local humoral immune response leads to the lymph nodes enlargement. However, there are limited reports regarding the LA manifestation worldwide, which is most likely observed in the nonsevere form of the disease [24]. In line with our report, the first case series of the positive subjects with confirmed CLA by MRI scan in Foch Hospital, France, was published several months after the SARS-CoV-2 pandemic [25]. Similarly, the report of the mediastinal LA in 15 COVID-19 patients admitted to the intensive care unit has also been described following the chest CT scan diagnosis in France [26]. Hilar LA is regularly observed during sarcoidosis, fungal and mycobacterial infections [27]. However, according to the recent report from the US Monmouth Medical Center, a 73-year-old Caucasian woman with typical symptoms of COVID-19 was diagnosed as an infected patient upon determining the positive result of the RT-PCR test. Notably, chest CT scan examination also displayed an atypical bilateral hilar LA, as well as focal consolidations, besides the multifocal subpleural ground-glass opacities [27].

Recently, a similar case series declared reactive LA in two patients who received the COVID-19 vaccine prior to radiological imaging [28]. The lymph node biopsy result confirmed the axillary LA as a vaccine-related post complication, which can mimic a metastasis, particularly in patients with a history of cancer [28].

Although a crosstalk between COVID-19 pathogenesis and the Herpesviruses coinfection is not well-established, some literature has demonstrated that the skin lesions induced by herpesvirus could occur during the SARS-CoV-2 infection. For instance, in Brazil, a 20-year-old female patient with a confirmed diagnosis of COVID-19 following RT-PCR test during two distinct episodes represented herpes simplex-induced lesions in the median of lower lip semi-mucosa [29]. Another case report also revealed a 70-years-old African–American woman with herpes zoster as a COVID-19 post complication, characterized by an erythematous patch, multiple vesicles and hemorrhagic crust on the left superior buttock [30].

In conclusion, we signified some atypical manifestations of COVID-19, including cervical and preauricular LAs, as well as a herpes-like lesion, following the confirmed positive diagnosis. Besides, given the possibility of lymph node involvement in COVID-19, a differential diagnosis should be considered in the case of patients referred to a physician with enlarged lymph nodes. Moreover, the etiology and pathophysiology of the COVID-19-induced lymph node enlargement can also be studied in detail. In more severe cases, the presence or absence of drug interactions should also be considered. Also, the relationship between the occurrence of LA and the demographic characteristics of individuals can be evaluated.

## Strength & limitation

Reporting such cases leads to notifying the physicians for COVID-19 rare symptoms identification; therefore, patients with a complaint of LA should also be suspected of COVID-19 morbidity. As a study limitation, no skin sampling and complementary tests were performed to detect the type of virus-induced lesions and to rule out other viral pathogens that involve in CLA pathogenesis.

**Executive summary**

- According to the recent evidence, it has been well-established that COVID-19 is taken into account as an extra-respiratory viral infection.
- Since inflammatory responses (cytokine storm) play a critical role in the COVID-19 progression, painful cervical and preauricular lymphadenopathies recently can also be listed as the uncommon COVID-19-induced symptoms even without respiratory tract involvement.
- Self-limited herpes-like lesions, for example, on the lower eyelid, can also be considered an atypical disease symptom.
